# Rubber hands in space: the role of distance and relative position in the rubber hand illusion

**DOI:** 10.1007/s00221-019-05539-6

**Published:** 2019-05-11

**Authors:** Andreas Kalckert, A. Treshi-Marie Perera, Yosindra Ganesan, Erika Tan

**Affiliations:** 1 School of Psychology, University of Reading Malaysia, Iskandar Puteri, Malaysia; 2 Faculty of Cognitive Science and Human Development, Cognitive Science Program, University Malaysia Sarawak, Kuching, Malaysia

**Keywords:** Rubber hand illusion, Ownership, Referral of touch, Multisensory integration, Peri-personal space

## Abstract

The rubber hand illusion (RHI) is a perceptual phenomenon in which participants experience ownership over a fake model hand through synchronous visuotactile stimulation. Several studies have shown that the illusion occurs only when both hands are in close proximity to each other. In the present study, we systematically examined the role of relative position (lateral, distal) and distance (13–75 cm) of the model hand (with respect to participants’ real hand) on illusion experience across both lateral and distal positions. Furthermore, we also compared different facets of the subjective illusion experience; the experience of the model hand being part of one’s body (i.e., ownership) and the perceptual fusion of vision and touch (i.e., referral of touch). In two experiments we observed indications for a stronger illusion experiences in distal compared to lateral positions of identical distances, indicating that the illusory effects may vary as a function of the relative position of the hand. Our results also showed that manipulations of distance differently modulated both facets of the illusion. While ownership was restricted to near distances, referral of touch sensations remained stable at farther distances. These results are interpreted in relation to variations in sensory weighting across different planes.

**Electronic supplementary material:**

The online version of this article (10.1007/s00221-019-05539-6) contains supplementary material, which is available to authorized users.

## Introduction

The rubber hand illusion (RHI; Botvinick and Cohen 1998) has provided a means of examining the experience of ownership in healthy participants. In this illusion, watching a fake model hand being stroked in synchrony with one’s real hand (that is out of view), creates the feeling that the rubber hand is a part of the body. This illusion is typically investigated with questionnaires, assessing the experience of ownership (e.g., I felt as if the rubber hand is my own hand) and/or the sensation of referral of touch (e.g., I felt the touch in the location I saw the rubber hand being touched; although see Tsakiris and Haggard 2005). A successful illusory experience is dependent upon a number of factors: (1) the synchronicity of the visual and tactile stimulation—so, asynchronous stimulation does not lead to the illusion (Botvinick and Cohen 1998; Tsakiris and Haggard 2005); (2) congruent anatomical orientation—hence, rotating the rubber hand with respect to participants’ real hand breaks the illusory experience (Ehrsson et al. 2004; Tsakiris and Haggard 2005) and (3) the spatial proximity between the two hands. If the distance between the two hands is greater than 30 cm the illusion is typically not experienced (Lloyd 2007; Preston 2013; Kalckert and Ehrsson 2014).

Armel and Ramachandran (2003) tested the effect of distance by placing the rubber hand 91 cm away from participants’ real hand and found a significant decrease in the illusory experience. In a more systematic investigation examining the role of distance between the real and rubber hands along a horizontal plane, Lloyd (2007) demonstrated that distances larger than 27.5 cm led to significant declines in the experience of the RHI (assessed through the referral of touch sensation). Preston (2013) also demonstrated reduced illusory experiences when a rubber hand was placed both far from the real hand as well as from the trunk. Alternatively, Zopf et al. (2010) found no difference in the experience of the illusion across distances of 15 and 45 cm using both subjective measures as well as in a cross-modal congruency task. Davies et al. (2013) compared the effect of distance between two variants of the RHI; the classical RHI and a non-visual self-touch illusion (STI) in which participants administered stimulation to the fake hand using their unstimulated hand. Their results indicated that whilst the classical RHI was robust against distance manipulations, the non-visual STI decreased as distance increased. Moreover, in a virtual hand illusion paradigm, Pritchard et al. (2016) demonstrated that an offset of 30 cm had no effect on experiences of ownership. Kalckert and Ehrsson (2014) compared the classical RHI to a moving RHI paradigm induced by finger movements (Tsakiris et al. 2006; Kalckert and Ehrsson 2012). They examined three different distances (12, 27.5, and 43 cm) along a vertical plane, however, in line with Lloyd (2007) demonstrated that the illusion decreased significantly with distances beyond 27.5 cm.

Successful illusory experiences across variants of the RHI (e.g., STI, moving RHI, etc.) might therefore depend upon the effective merging of sensory information across different modalities, leading to higher order experiences of ownership. Distance effects have provided evidence for a spatial boundary in the experience of the RHI and supports the idea that the illusion might be based on processes akin to multisensory integration that follows basic temporal and spatial rules (Stein and Stanford 2008; Ehrsson 2012). Multisensory and sensorimotor integration are best explained through Bayesian statistics (see e.g., Ernst and Bülthoff 2004; Körding et al. 2006, 2007; Bays and Wolpert 2007) which show that the weights assigned to different pairs of sensory stimuli can change and that the nervous system flexibly integrates this sensory information depending on the context. Ernst and Banks (2002) studied the integration of haptic and visual cues in a size-estimation task and found that our perceptual system relies predominantly on cues with lower variances or in other words cues providing more precise estimates (see also Newport et al. 2002). Additionally, Van Beers et al. (2002) investigated the relationship between visual and proprioceptive cues in a localisation task with an adaptation paradigm. They found both these cues to be differently weighted depending on the plane: in-depth visual adaptation was significantly larger than proprioceptive adaptation, with the opposite observed in the horizontal plane. The nervous system therefore, directly assigns different precisions to vision and proprioception along the different planes suggesting that the integration of visual and somatosensory input can be directly influenced by the spatial configuration of stimuli (see also Snijders et al. 2007).

Given that the RHI relies on the integration of visual, tactile and proprioceptive cues, whether or not the experience of the illusion might vary with respect to specific directions in space remains to be examined. As most RHI experiments are performed along a horizontal layout with the rubber hand being placed medially with respect to participants’ real hand (e.g., Botvinick and Cohen 1998; Tsakiris and Haggard 2005; Lloyd 2007), whether or not the results are a consequence of this specific spatial configuration can be questioned and forms the basis of the current investigations. Furthermore, evidence for the role of spatial distance between the real and rubber hands on experiences of the illusion appear mixed. Hence, at present unequivocal conclusions that support the experience of the RHI to be driven by spatially congruent multisensory mechanisms cannot be drawn. Therefore, we investigated the role of position and distance on the RHI by directly comparing subjective illusory experiences along horizontal and distal planes. We conducted two experiments; in Experiment 1 we tested a close distal position (13 cm) and compared this to the same as well as a further distance of 38 cm along a horizontal plane. In Experiment 2 three different distances (13, 38 and 75 cm) along the distal plane were compared. If the experience of the classical RHI is mediated by differences in sensory weighting across different positions (Van Beers et al. 2002), then we expect to see differences in illusion susceptibility across both lateral and distal positions. The RHI has been found to be reliably induced at distances below 20 cm (see e.g., Tsakiris and Haggard 2005; Lloyd 2007; Kalckert and Ehrsson 2014) with distances of 38 or 75 cm not permitting the illusion (see e.g., Lloyd 2007; Kalckert and Ehrsson 2014; Guterstam et al. 2013). Therefore, we expected a decrease in illusion experiences as distance increased along both horizontal and distal planes. The illusion is therefore, predicted to be present at the near distance of 13 cm, but not at distances of 38 cm or 75 cm, respectively. Our results suggested that the relative distance between the real and model hands had an effect on the RHI in both planes, and also that the distal plane showed higher subjective illusion experiences compared to the horizontal plane. Furthermore, we also observed differences in ownership and referral of touch-related scores across the two positions and distances.

## Materials and methods: Experiment 1

### Participants

Fifty-five naive participants (31 female; mean age 20.53 years; SD 4.19) participated in the study. Written informed consent was obtained from all participants prior to participation. All participants had normal or corrected vision, and none reported any sensory deficits. The study was approved by the University of Reading Research Ethics Committee and has been conducted in accordance to the declaration of Helsinki. Participants received a monetary compensation for their participation.

### Procedure

**Fig. 1:**
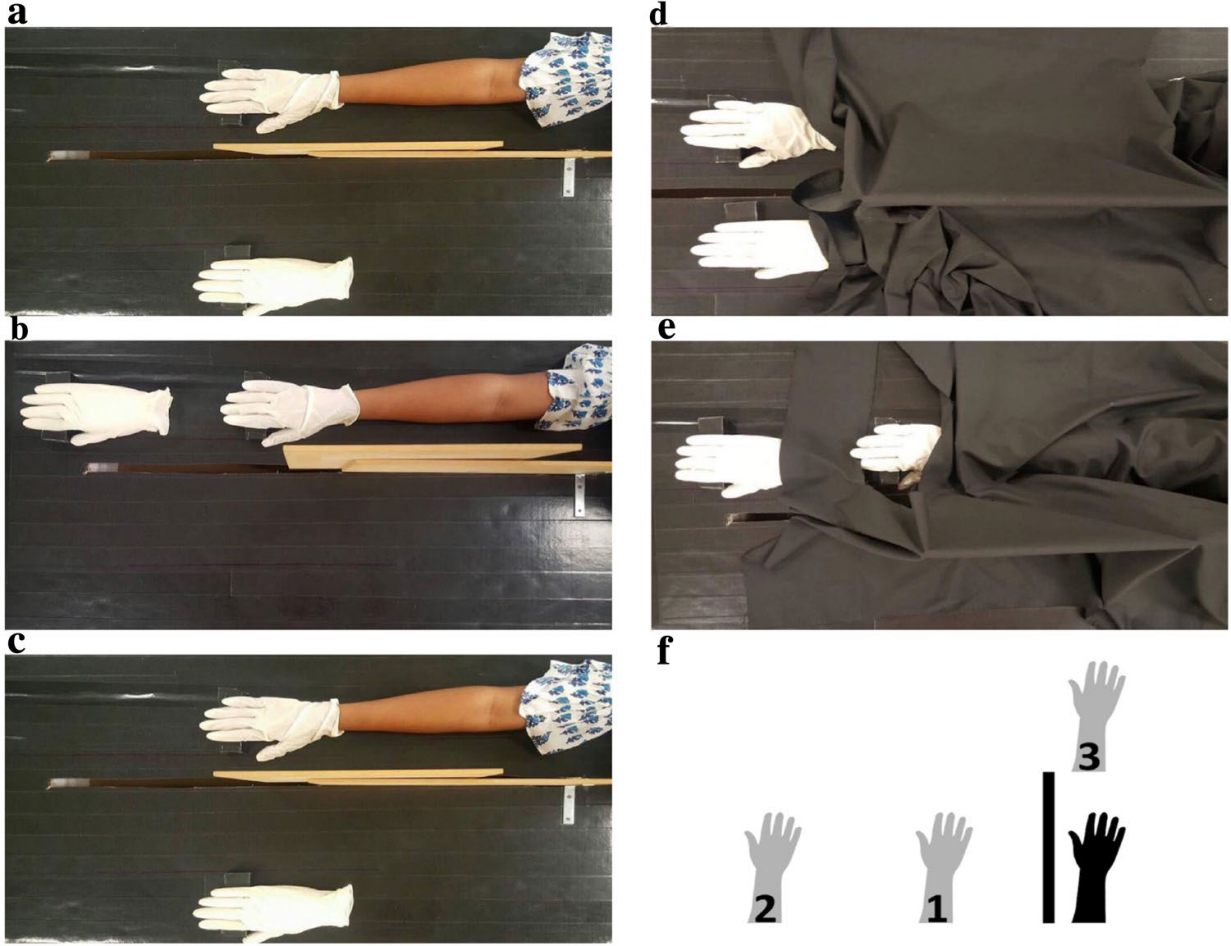
Illustration of the setup in Experiment 1: **a** lateral-near (13 cm), **b** distal-near (13 cm) and **c** lateral-far (38 cm). A black cloth covered both arms as shown in **d** and **e** in all conditions. **f** Schematic diagram of all conditions

Participants were comfortably seated in front of a table on a height-adjustable chair with their right arm maximally stretched out and placed on the table. The right arm was positioned such that it was occluded from view by a wooden board and the right shoulder was covered with a black cloth. The fingers were placed comfortably on a wooden cube to elevate them and make the fingers more visible at the distal position. Participants sat close to the table ensuring that their upper body leaned close against the table. This prevented participants from moving and ensured no arm movement. A life-size model hand was then placed in one of three positions: distal-near, lateral-near (both 13 cm), and lateral-far (38 cm) (see Fig. 1). The lateral distances were measured from the participants’ thumb joint, while the distal distance was measured from the tip of the index finger. In each position, participants went through both synchronous and asynchronous blocks of trials. During synchronous stroking, simultaneous brush strokes were applied to the proximal joint of the index finger at a frequency of 1 Hz, paced by an auditory signal presented to only the experimenter. A temporal delay of approximately 500 ms was applied to brush strokes between both hands during asynchronous stroking. Each stimulation condition lasted 90 s, and single and double brush-strokes (i.e., two rapid successive strokes) were applied in a pseudo-random sequence to introduce variability and minimise effects of predictability of brush strokes. The order of the positions as well as the synchronous/asynchronous conditions was randomised across participants. The experimenter’s hands were also not visible to participants at any point.

Following stimulation participants responded to a series of eight randomly presented questionnaire statements on a computer screen placed on a table to participants’ right. These included two ownership items and two referral of touch items—both reflecting illusion experience (statements 1–4, see Table 1), as well as four control statements that did not tap into any illusion-related aspects (statements 5–8 see, Table 1). Participants made ratings on a seven-point Likert-scale in which − 3 indicated strongly disagree, 0 indicated neutral and + 3 strongly agree. Statements were adopted from previous RHI experiments (Botvinick and Cohen 1998; Longo et al. 2008). Following the experiment, participants were asked to estimate the length of their arm by placing two pins on a foam board, after which their actual arm length was measured. This allowed us to examine whether there might be a potential link between illusion experience in the distal position and participants’ self-perceived and actual arm length (see suppl. Data).

**Table 1:** Statements used in the questionnaire

Ownership	
1	I felt as if the model hand was part of my body
2	I felt as if the model hand was my hand
Referral of touch	
3	I felt the touch of the brush in the location where I saw the model hand being touched
4	I felt as if the touch was caused by the brush touching the model hand
Control	
5	I felt as if my real hand was turning rubbery
6	It felt as if I had no longer had a right hand, as if my right hand had disappeared
7	It seemed as if the touch I was feeling came from somewhere between my own right hand and the model hand
8	It seems as if I had more than one right hand

## Results: Experiment 1

Overall illusion scores and control scores were calculated by averaging the four illusion-related statements (i.e., ownership and referral of touch) and control statements for each condition separately. Additionally, sub-scores for ownership and referral of touch were computed by separately averaging statements 1 and 2 (ownership) and 3 and 4 (referral of touch). In line with previous studies, we considered a participant with an average illusion score of ≥ 1 as an illusion responder (Petkova and Ehrsson 2009) and a grouped median ≥ 1 to affirm the overall illusion experience (i.e., combined ownership and referral of touch scores) as well as for each independent sub-score (Kalckert and Ehrsson 2012). To establish the presence of the illusion at each position, we compared overall illusion scores between the synchronous and asynchronous conditions, as well as overall illusion and control scores in the synchronous condition using Wilcoxon signed-rank tests. The role of distance and position on illusion susceptibility was examined by comparing illusion scores in synchronous conditions using a Friedman test. Effect sizes were computed with the following formula *r* = *z*-score/(√*n*). All reported results are two-tailed, and all tests are based on a priori hypotheses unless otherwise stated.

### Lateral-near

**Fig. 2:**
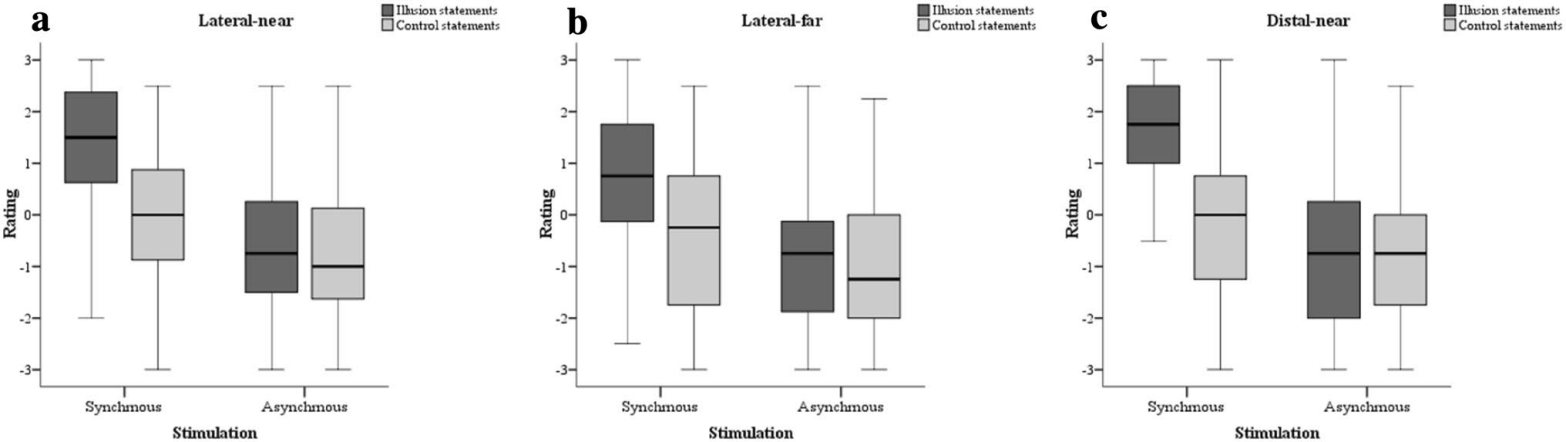
Box and whisker plots for illusion and control scores during synchronous and asynchronous stimulation. **a** Lateral-near, **b** lateral-far and **c** distal-near

Participants had significantly higher illusion scores in the synchronous (*M*: 1.5), compared to the asynchronous condition (*M*: − 0.75; *Z* = − 5.733, *p* < 0.001; *r* = 0.55). A significant difference was also observed between illusion and control statement scores (*M*: 0) in the synchronous condition with illusion statements being higher (*Z* = − 5.579, *p* < 0.001; *r* = 0.53; see Fig. 2a).

### Lateral-far

Participants had low positive ratings to illusion statements in the synchronous condition (*M*: 0.75), and negative ratings in the asynchronous condition (*M*: − 0.75). This difference was significant (*Z* = − 5.083, *p* < 0.001; *r* = 0.49). Ratings to illusion statements were significantly higher than the control statements in the synchronous condition (*M*: − 0.25; *Z* = − 4.641, *p* < 0.001; *r* = 0.44; see Fig. 2b).

### Distal-near

Ratings to illusion statements were high in the synchronous condition (*M*: 1.75), but not in the asynchronous condition (*M*: − 0.75). This difference was significant (*Z* = − 5.671, *p* < 0.001; *r* = 0.54). Participants also gave significantly higher ratings to illusion statements compared to the control statements in the synchronous condition (*M*: 0; *Z* = − 6.194, *p* < 0.001; *r* = 0.59; see Fig. 2c).

### Comparison of illusion scores under synchronous stimulation across the three positions

**Fig. 3:**
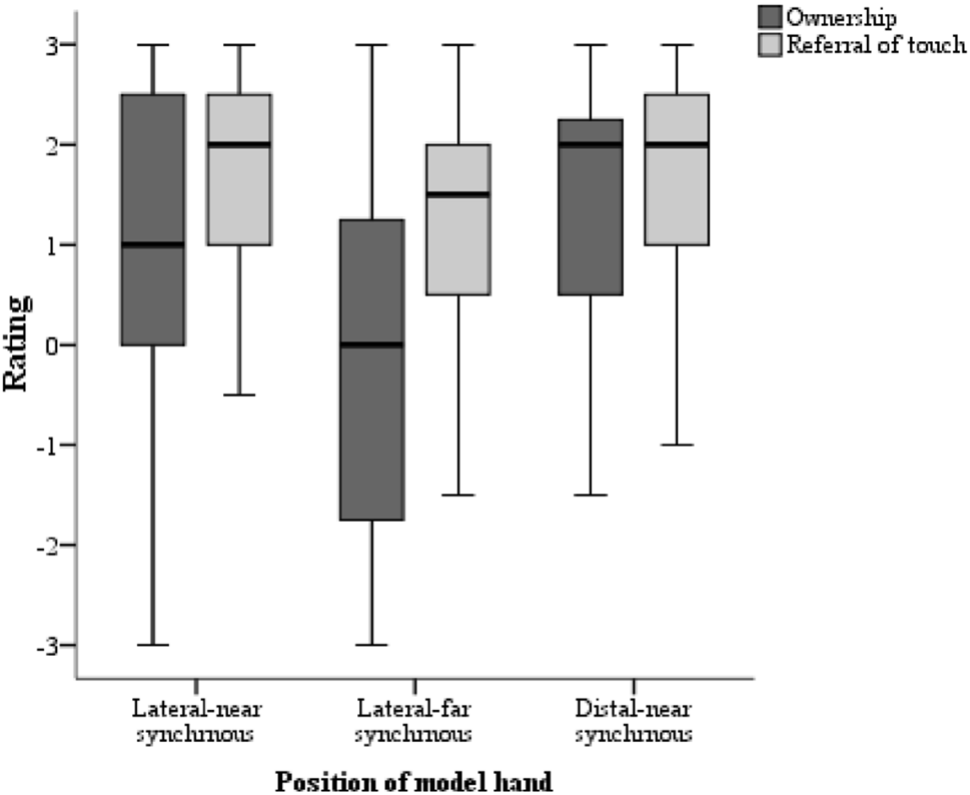
Box and whisker plots for ownership and referral of touch scores across the three synchronous conditions

We compared illusion scores of the three synchronous conditions and found significant differences in illusion ratings (Friedman: *χ*^2^ [2, *N* = 55] = 16.820, *p* < 0.001; see Fig. 3). Subsequent post hoc pairwise comparisons found illusion scores in both the distal-near and lateral-near positions to be significantly different from the lateral-far position (distal-near vs. lateral-far: *Z* = − 3.801, *p* < 0.001; *r* = 0.36; and lateral-near vs lateral-far: *Z* = − 3.401, *p* = 0.001; *r* = 0.32). The difference between the lateral-near and the distal-near positions was not significant (*Z* = − 1.944, *p* = 0.052; *r* = 0.18).

### Ownership versus referral of touch-related illusion questions

Ratings to these two subcategories of statements were compared across all three distances in the synchronous condition. Median ownership and referral of touch scores were as follows; lateral-near (ownership = 1, referral of touch = 2), lateral-far (ownership = 0.0, referral of touch = 1.5) and distal-near (ownership = 2, referral of touch = 2). In all synchronous conditions, the referral of touch scores were higher than ownership scores (lateral-near: *Z* = − 3.439, *p* = 0.001; *r* = 0.33; lateral-far: *Z* = − 5.334, *p* < 0.001; *r* = 0.51; distal-near: *Z* = − 2.744, *p* = 0.006; *r* = 0.26; see Fig. 3).

Next, two Friedman tests on ownership and referral of touch scores were independently conducted. A significant main effect for ownership scores was observed: *χ*^2^ [2, *N* = 55] = 20.160, *p* < 0.001. Subsequent pairwise comparisons indicated significant differences between the lateral-far position compared to both the lateral-near (*Z* = − 3.837, *p *< 0.001, *r* = 0.37) and distal-near (*Z* = − 4.154, *p* < 0.001, *r* = 0.40) conditions. The difference between the lateral-near and distal-near positions was not found to be significant (*Z* = − 1.653, *p* = 0.098). No significant differences for referral of touch scores were found across the conditions (Friedman: *χ*^2^ [2, *N* = 55] = 5.683, *p* = 0.058), and no subsequent pairwise comparisons were performed.

## Discussion: Experiment 1

In line with previous studies (e.g., Lloyd 2007), the current results demonstrated significantly lower illusion ratings in the lateral-far position compared to the lateral-near position thus indicating a spatial boundary in the experience of the RHI along horizontal planes. Interestingly, the findings also indicated the presence of the RHI in the distal-near position. We propose that synchronous visuotactile stimulation of the model arm along the distal axis may have functionally extended peripersonal space boundaries thus enlarging receptive field size corresponding to the hand. This expansion of receptive field size might have permitted synchronous visuotactile processes to drive illusory experiences leading to higher order feelings of ownership. Indeed, synchronous somatosensory input on the hand coupled with auditory (or visual stimulation) in far-space has recently been shown to extend peripersonal space in a manner similar to that following tool use (Serino et al. 2015). The operation of such mechanisms along the distal (as opposed to the horizontal) plane can be explained with regards to the optimal integration model (Van Beers et al. 2002) which proposes visual and proprioceptive sensory weighting to vary as a function of direction. Such differences might have influenced the integration of sensory information on which the illusion relies on leading to the experience of the illusion at this position (Lackner and DiZio 2000).

Given the presence of the illusion in the distal position, we conducted a follow-up study to precisely delineate the role of increasing distal distances between the real and model hand on the RHI. As we observed a trend towards a difference between lateral and distal-near positions in Experiment 1, the lateral-near position was again included in Experiment 2.

## Experiment 2

### Participants

Forty-four naive participants took part in Experiment 2 (27 female; mean age 24.27; SD = 7.06). All participants provided written informed consent, had normal or fully corrected vision and reported no sensory deficits.

## Materials and methods: Experiment 2

**Fig. 4:**
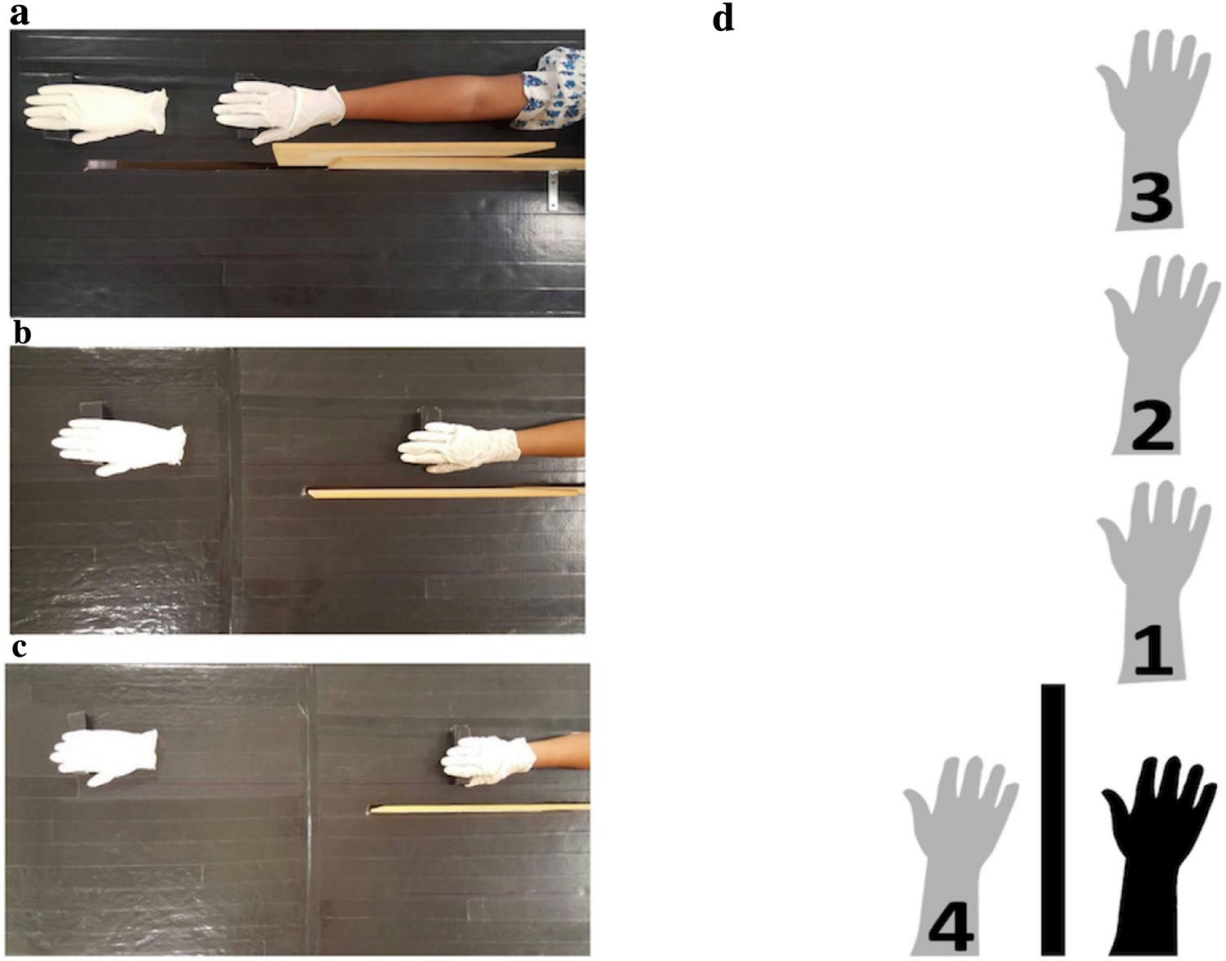
Illustration of the setup for Experiment 2 distal positions: **a** distal-near (13 cm), **b** distal-far (38 cm), **c** distal-very-far (75 cm), **d** schematic diagram of all conditions including lateral-near

The materials used, and experimental procedures were identical to Experiment 1. Both synchronous and asynchronous stimulation conditions were tested across all three distal positions (see Fig. 4). The synchronous lateral-near position was included to enable comparisons between the near positions of both planes (thus totalling to seven conditions). The distance in both near positions, far and very far positions were 13, 38 and 75 cm, respectively. All conditions were randomised and balanced across participants.

## Results: Experiment 2

Data collation and analyses were identical to Experiment 1.

### Distal-near

**Fig. 5:**
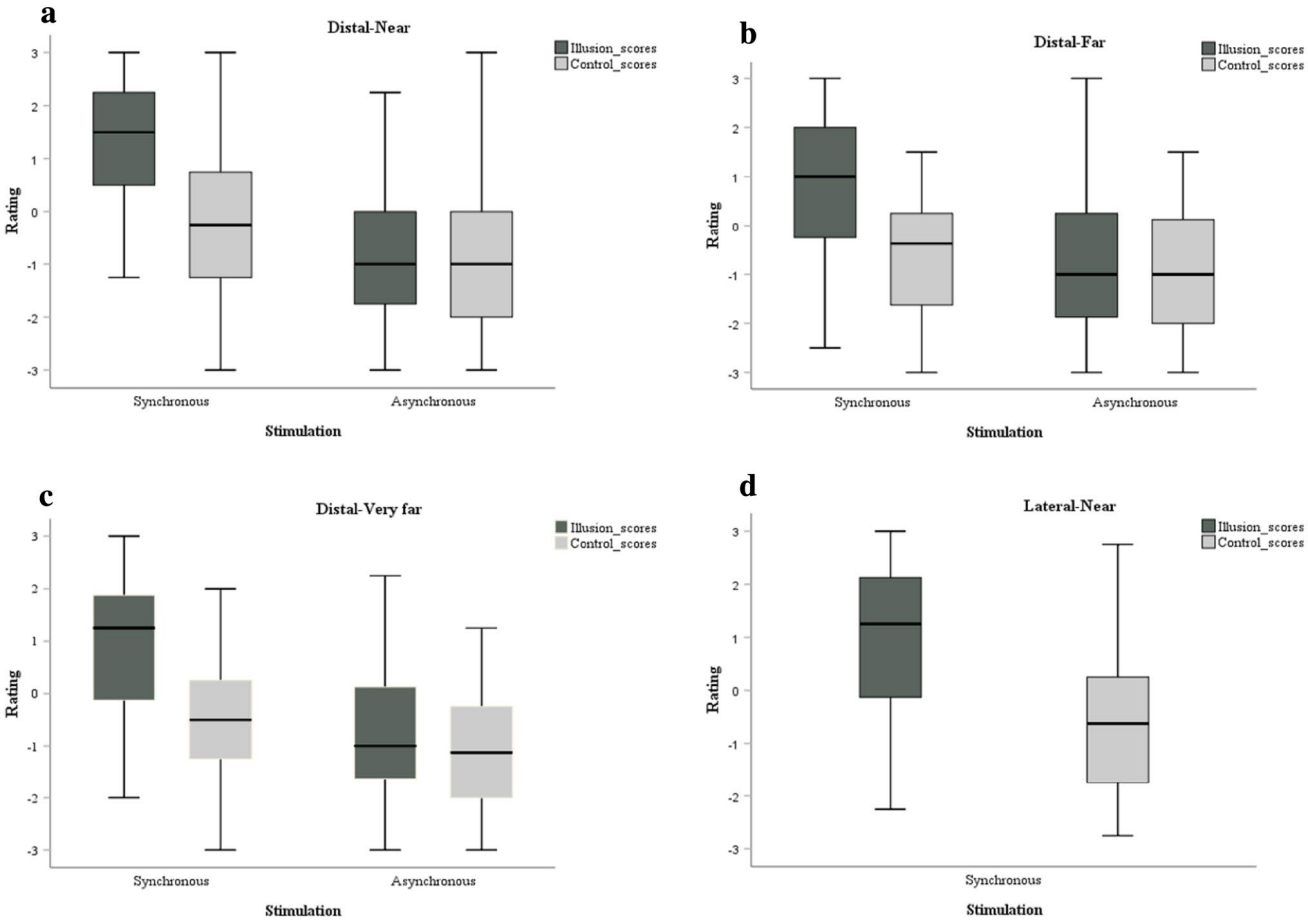
Box and whisker plots for illusion and control scores during synchronous and asynchronous stimulation. **a** Distal-near, **b** distal-far, **c** distal-very-far and **d** lateral-near (synchronous)

Overall illusion ratings in the synchronous condition (*M*: 1.5) were higher than the asynchronous condition (*M*: − 1; *Z* = − 5.468, *p* < 0.001; *r* = 0.58). Overall illusion ratings were also significantly higher compared to control statement scores (*M*: − 0.25) in the synchronous condition (*Z* = − 5.527, *p* < 0.001; *r* = 0.59, see Fig. 5a).

### Distal-far

Ratings to the illusion statements were positive in the synchronous condition (*M*: 1) and negative (*M*: − 1) in the asynchronous condition. This difference was significant (*Z* = − 4.773, *p* < 0.001; *r* = 0.51). Here again, significantly higher ratings to the illusion statements compared to the control statements (*M*: − 0.38; *Z* = − 4.860, *p* < 0.001; *r* = 0.52, see Fig. 5b) were observed in the synchronous condition.

### Distal-very far

This condition also demonstrated positive ratings (*M*: 1.25) for illusion scores in the synchronous condition compared to the asynchronous condition, in which overall illusion scores were negatively rated (*M*: − 1). This difference was significant (*Z* = − 4.792, *p* < 0.001; *r* = 0.51). Ratings to illusion statements were significantly higher than control statements (*M*: − 0.5; *Z* = − 4.792, *p* < 0.001; *r* = 0.51, see Fig. 5c) in the synchronous condition.

### Lateral-near

Participants gave significantly higher ratings for illusion (*M*: 1.25) compared the control statements (*M*: − 0.63) in the synchronous condition (*Z* = − 5.181, *p* < 0.001; *r* = 0.55).

### Comparison of illusion scores under synchronous stimulation across positions

A significant main effect for overall illusion ratings were observed across the three distal conditions and lateral-synchronous condition (Friedman: *χ*^2^ [3, *N* = 44] = 18.754, *p* < 0.001). Post hoc pairwise comparisons demonstrated significant differences in illusion scores between the distal-near and distal-far position (*Z* = − 3.478, *p* = 0.001; *r* = 0.37), the distal-near and distal-very far position (*Z* = − 3.274, *p* = 0.001; effect size *r* = 0.35) as well as the distal-near and lateral-near position (*Z* = − 2.758, *p* = 0.006; *r* = 0.29). No other comparisons were significant at a Bonferroni corrected significance level of *p *= 0.008.

### Ownership versus referral of touch-related illusion questions

**Fig. 6:**
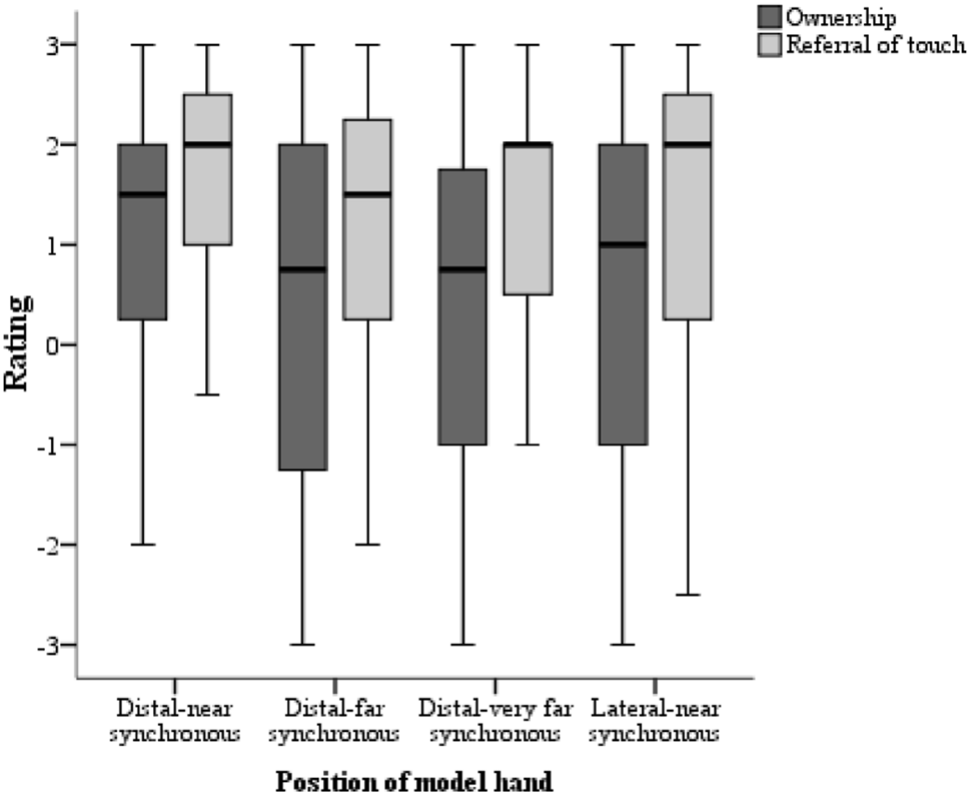
Box and whisker plots for ownership and referral of touch scores across all synchronous conditions

Median ownership and referral of touch scores across the different conditions were as follows; distal-near (ownership = 1.5, referral of touch = 2), distal-far (ownership = 0.75, referral of touch = 1.5), distal-very far (ownership = 0.75 referral of touch = 2) and lateral-near (ownership = 1, referral of touch = 2). In line with Experiment 1, we compared ownership and referral touch scores and observed significantly higher referral of touch scores in the distal-near (*Z* = − 3.433, *p *= 0.001; *r* = 0.37), distal-far (*Z* = − 4.125, *p *< 0.001; *r* = 0.44), distal-very far (*Z* = − 4.455, *p* < 0.001; *r* = 0.47), and lateral-near (*Z *= − 3.559, *p* < 0.001; *r* = 0.38) conditions (see Fig. 6).

A Friedman test conducted across the distal positions and lateral-near position showed a main effect for ownership scores (*χ*^2^ [3, *N* = 44] = 12.42, *p* = 0.006). Follow-up, pairwise comparisons demonstrated significant differences between the distal-near and the two farther distal positions; (distal-near vs distal-far, *Z* = − 2.817, *p* = 0.005, *r* = 0.30 and distal-near vs distal-very far, *Z* = − 2.670, *p* = 0.008, *r* = 0.28). No significant differences between the distal-far and distal-very far positions (*Z* = − 0.115, *p* = 0.908) as well as between the distal-near and lateral-near positions (*Z* = − 1.781, *p* = 0.075) were observed (Bonferroni corrected *p *= 0.008). A second Friedman test on referral of touch scores also revealed a significant difference across the conditions (*χ*^2^ [3, *N* = 44] = 8.021, *p* = 0.046). Follow-up pairwise comparisons indicated significant differences between distal-near position and distal-far (*Z* = − 2.982, *p* = 0.003, *r* = 0.32) as well as the distal-near and lateral-near positions (*Z* = − 2.771, *p* = 0.006, *r* = 0.30) positions. Significant differences were also not observed between the distal-near vs. distal-very far (*Z* = − 1.903, *p* = 0.057) and distal-far vs. distal-very far conditions (*Z* = − 0.837, *p* = 0.43; Bonferroni corrected significance value of *p *= 0.008).

## Discussion: Experiment 2

Experiment 2 expanded upon the findings of Experiment 1 by examining the modulatory role of increasing distal distances on the RHI. Our findings provided evidence for a stronger illusion at the distal-near position compared to a lateral position of similar distance. This supports our interpretation of a direction-dependant weighting of visuoproprioceptive signals that might interact to drive illusory experiences. Furthermore, a decrease in overall illusion experience, along increasing distal distances was observed, thus providing evidence for the role of distance on the RHI.

Close examination of the illusion subcategories demonstrated that ownership scores decreased along increasing distal distances, with the strongest experience of ownership only observed at the distal-near position. No significant difference in ownership between the distal-far and distal-very far positions was observed, thus indicating a spatial boundary for ownership along this plane. Furthermore, in line with Experiment 1, no significant difference between the lateral and distal-near positions was also observed, suggesting that ownership might be restricted to peri-hand space along both distal and lateral planes. Referral of touch, on the hand, was found to be stronger in the distal-near compared to the distal-far and lateral-near positions. While this provides evidence for a preference of visuotactile integrative processes at peri-hand spaces along the distal planes; the fact that referral of touch scores still remained high (i.e., ≥ 1) even at the farther distal distances should not be ignored. Indeed, as with Experiment 1, referral of touch scores remained significantly higher than ownership scores across at all distances. The overall response patterns may therefore, suggest that although stronger integrative processes may occur at near-spaces, visuotactile process might still be at operation even at farther spaces from a body.

## General discussion

The present study measured the effect of distance and position on the rubber hand illusion. We observed higher illusion scores in distal positions compared to lateral positions when distance was kept the same. Our results also showed that distance generally affects the illusion, and provides a detailed account of the role of distance on RHI experiences. While increasing distances led to weaker subjective illusory reports, this effect was specific to the subjective component of the illusion assessed, i.e., ownership experiences decreased when the model hand was placed more than 27 cm away while referral of touch sensations were rated high (i.e., ≥ 1) at all distances.

Our observation of a general distance effect is in agreement with previous studies showing that as distance increases, the illusion significantly decreases (Lloyd 2007; Kalckert and Ehrsson 2014 but see Zopf et al. 2010). Hence, successful illusion experiences depend upon the close proximity between the model hand and participants’ own hand. Most RHI studies place the rubber hand medially with respect to participants’ real hand (i.e., Lloyd 2007) as introduced in the original study by Botvinick and Cohen (1998). Indeed, very few studies used different arrangements, and these were motivated by practical constraints such as in functional imaging studies (see e.g., Ehrsson et al. 2004). These studies however, did not directly address the role of position on the illusion. The current study therefore adds to the previous research by showing that distance affects the RHI not only in the lateral (Lloyd 2007; Preston 2013) and vertical planes (Kalckert and Ehrsson 2014), but also, along a distal plane.

We observed a stronger illusion at the distal-near position compared to the lateral-near position of 13 cm (see “Experiment 2”). Such a finding was unexpected given that at this position the model hand was placed beyond reaching space (see Fig. 1b). Although speculative at the time, we offer two potential explanations for the experience of the RHI at the distal-near position. Previous theories have generally argued that bodily illusions like the RHI are restricted to peripersonal space surrounding the body/hand (Brozzoli et al. 2014; Makin et al. 2008). One possibility can be linked to errors made in judgements of body-part size such as the hand (e.g., Longo and Haggard 2012). If perceived arm length was overestimated, participants may not have been aware of the fact that the rubber hand was placed beyond reach, thus reducing conflict between the seen (model) hand location and the felt (real) hand location. Alternatively, it is also possible that placement of the model arm in the lateral-near position might have introduced a muscular-skeletal rotation of the shoulder and elbow which might have signalled a visuoproprioceptive conflict, not present in the distal position. The absence of such conflicting sensory signals could have led to stronger illusory experiences in the distal position.

At a mechanistic level, differences between the distal and lateral positions can be related to alterations in the perceptual processes underlying the illusion. The multisensory representation of the space surrounding the hand is coded for by visuotactile bimodal neurons found in the parietal and premotor regions as well as the putamen (Graziano and Botvinick 2002; Stein and Stanford 2008). These bimodal neurons have receptive fields that are restricted to peripersonal space (Rizzolatti et al. 1981). Hence as described in the discussion of Experiment 1, the experience of the illusion along the distal plane could be linked to an expansion of visuotactile receptive fields corresponding to the hand as a result of synchronous visuotactile stimulation (Serino et al. 2015). Moreover, the study by Van Beers et al. (2002) has shown that vision and proprioception are differently weighted along horizontal and distal planes (see also Van Beers et al. 1999). In the distal plane vision adapts more than proprioception, thus proprioception is estimated to be more precise. Along the horizontal plane proprioception adapts more than vision. Classical concepts of the RHI define the illusion as a triangulation of vision, tactile, and proprioception input with their respective alignment (Botvinick and Cohen 1998; Makin et al. 2008), constrained by top-down mechanisms (Tsakiris and Haggard 2005; Tsakiris et al. 2010). The observation by Van Beers et al. (2002) could be interpreted in a way that either the lateral position could benefit in terms of the illusion experience (i.e., the higher proprioceptive adaptation may allow an easier alignment to the visuotactile event on the rubber hand) or even the distal position (i.e., visual estimation of hand position may adapt more to the rubber hand in sight, and may allow the visual integration to be estimated at the rubber hand). Interestingly, the findings seemed to suggest that the distal position permitted stronger illusion experiences. However, the study by Van Beers and colleagues investigated the combination of vision and proprioception without the visuotactile component, hence, at present, the precise roles of the perceptual weights on the integrative processes underlying the RHI can only be speculated. Future studies should address how different sensory cues relevant to the RHI are weighted and integrated across different distances and planes.

At first glance, our data may suggest that an illusion is present at all distances, with high illusion scores even at a 75 cm distance. This is in contradiction to previous findings, which found that the illusion declined with increasing distances (Lloyd 2007; Kalckert and Ehrsson 2014). We must however, be more specific when interpreting our results. Our questionnaire contained statements relating to both sensations of ownership and the experience of referral of touch. Therefore, when independently examining these statement categories, a more specific pattern was observed. Whilst ownership scores declined with increasing distances, referral of touch scores remained high across all distances. Therefore, while synchronous visuotactile stimulation could have led to the perceptual fusion of touch (or experiences of referral of touch) through peripersonal space expansion (Serino et al. 2015), under certain conditions (such farther spatial distances) this might not lead to higher order experiences such as ownership. Indeed, ownership experiences are constrained by top-down factors (such as knowledge about the appearance and shape and size of a body part) and violations of such top-down knowledge have been found to breakdown ownership despite synchronous visuotactile stimulation (Tsakiris and Haggard 2005; Tsakiris 2010). Hence, as previously stated although ownership can be altered as a result of the plasticity of peripersonal space boundaries (along the distal plane at least), there are still constraints to the development of ownership at more extreme distances. This could be due to weaker sensory processing at farther distances that may not have been sufficient to induce ownership. Whether or not other perceptual processes such as longer durations of visuotactile stimulation or changes to stimulation frequency/intensity may lead to ownership at farther distances in extrapersonal space nevertheless warrants further investigation.

Alternatively, it is also possible that the mere sight of the model hand, might have influenced the integrative processes that led to referral of touch sensations. A study by Guterstam et al. (2013) tested the invisible hand illusion (a variant of the RHI in which there is no model hand) and did not observe an illusion at a distance of 75 cm. It was suggested that this could be due to such illusions relying on multisensory rules such as synchrony and distance. In the current setup however, participants viewed a model hand in front of them. Indeed, previous studies have shown that viewing the body alters tactile processing (Haggard et al. 2007; Longo et al. 2011) and visuotactile integration (Mirams et al. 2010). Furthermore, it should also be noted that in our setup, the placement of the model hand created the impression of an extension of arm length, as opposed to a misalignment in its position. In relation to this, studies inducing bodily illusions in virtual reality have shown that participants can perceive unrealistic arm elongations (Kilteni et al. 2012). Furthermore, when viewing only a rubber hand, without ever touching the hands, participants showed increased expectations of an approaching tactile stimulus, as if the sensory predictions are centred on the seen rubber hand (Ferri et al. 2013). It is therefore, possible that that the visual presence of a hand-shaped object might have permitted visuotactile integrative processes at farther distances in space leading to referral of touch experiences.

Our interpretation of visuotactile integration in the far distances can be supported by single cell studies on bimodal neurons. These cells fire in response to visual and tactile stimuli approaching or touching the hand (Rizzolatti et al. 1981; Hyvärinen 1981; Duhamel et al. 1998; Graziano and Gross 1994) making them suitable neural substrates underlying the RHI and the perception of our body more generally (Ehrsson 2012). Although the role of bimodal neurons in integrative processes are thought to be restricted to the space immediately surrounding the hand (Brozzoli et al. 2014), there is evidence for visuotactile integration at farther distances. For example, Graziano and Gross (1994) reported that while premotor and parietal neurons preferred stimulation at immediate distances (< 20 cm) surrounding a body part, a significant proportion of premotor neurons also reacted to stimuli up to 1 m (22%), or even beyond (39%). For parietal neurons this proportion was even greater: 42% responded to distances of up to 1 m, and 16% to distances beyond 1 m. Interestingly this study also reported that some of these neurons are relatively flexible in their receptive field size. A neuron can show the strongest response to a distance of 20 cm but can respond (in a relatively weaker fashion) to a visual stimulus as far away as 2 m. Moreover, in a later study Graziano et al. (2000) found activity in approximately 1/3 of parietal bimodal neurons when a monkey viewed a fake arm in place of its real arm. Importantly, this effect was specific to the presence of a congruent and realistic arm shaped object. This suggests that in addition to merely responding to visual and somatosensory signals, properties of some parietal bimodal neurons may also drive mechanisms underlying the incorporation of objects into the body representation (Graziano et al. 2000).

The fact that ownership and referral of touch experiences reflects different aspects of the RHI experience has implications for current research practices, as most RHI studies either incorporate combinations of both kinds of statements or use these interchangeably as proxies of the illusion. The findings by Lloyd (2007) and Kalckert and Ehrsson (2014) suggest that the illusion significantly declines at distances beyond 27 cm, however they used different statements to measure the illusion. Although this discrepancy cannot be resolved, we highlight the need for a closer look at the specific response patterns on both facets of the illusion. Our results indeed demonstrate that manipulations of the RHI could differently affect these two experiences (e.g., permitting a referral of touch, but not ownership). This distinction also poses practical (i.e., interpretation of ratings) and theoretical implications (i.e., relationship between the processes underlying visuotactile fusion versus ownership experiences). Future research needs to address the relationship between ownership and referral of touch within the RHI, and how these are individually expressed in specific experimental situations. One hypothesis is that the fusion of visuotactile stimuli is the initial step in the illusion process, and that further processes then convert this basic perceptual integration into higher order experience of ownership (see Makin et al. 2008; Tsakiris et al. 2010).

In conclusion, the present results extend previous literature on the role of distance in RHI experiences by demonstrating differences in illusion strength across lateral and distal planes. This might be a result of direction-dependant variations in the illusion-related sensory weighting. The findings may therefore, suggest how ownership experiences extend beyond the physical and anatomical constraints of the body. Finally, we also found two subjective components of the illusion (ownership and referral of touch) to be differently influenced by manipulations of spatial disparity, and thus raises theoretical and methodological concerns regarding what constitutes the experience of ownership and the establishment of the rubber hand illusion.

## Electronic supplementary material

Below is the link to the electronic supplementary material.

Supplementary material 1 (DOCX 4867 kb)
