# Supplementary material for: Rubber hands in space: the role of distance and relative position in the rubber hand illusion
Source: Exp Brain Res. 2019 May 11;237(7):1821–32. doi: 10.1007/s00221-019-05539-6 (PMC6584242; doi:10.1007/s00221-019-05539-6)
Supplement: Supplementary file 1 — Supplementary material 1 (DOCX 4867 kb) [file 221_2019_5539_MOESM1_ESM.docx]

Supplementary data

Rubber hands in space – the role of distance and relative position in the rubber hand illusion

Authors: Andreas Kalckert*, Andrea Treshi-Marie Perera*, Yosindra Ganesan, Erika Tan

*shared first authorship

# Experiment 1 - Effect of perceived arm length

We also examined the existence of a potential link between participants’ arm length and illusion experience in the distal-near position. Participants’ arm length on average was 71.60 cm (SD = 1.94). The subjective arm length was 77.70 cm, indicating an overestimation in perceived length of the arm.

Percentage of overestimation/underestimation of arm length was then calculated using the following formula (real length / perceived length) * 100. On average, participants perceived their arm to be 7.9% longer. No significant correlation was however, seen between arm length and illusion score in the distal-near (synchronous) condition (Spearman’s rho: -0.63, n = 55, *p* = 0.649).

As mentioned in the main text, there is evidence for general misperceptions in body part size (Longo & Haggard, 2012) which in the case of the current study might have led to the model hand seeming closer to the boundary of reaching space than in reality. Nevertheless, as perceived arm length was only assessed at the end of the experiment whether or not the observed overestimation in arm length was a result of the experimental condition or general propensities towards misperceiving body part size cannot be ascertained for certain.

# Experiment 1 - Ownership vs referral of touch related questions

As stated in the main text, our subjective questionnaire included a combination of both ownership and referral of touch statements. We examined the link between the two statements using Spearman’s rho correlation analyses. Both ownership and referral of touch scores were highly correlated at all positions in the synchronous condition (see Fig S1): distal-near (Spearman’s rho: 0.658, n = 55, p < .001), lateral-near (Spearman’s rho: 0.667, n = 55, p < .001), and lateral-far (Spearman’s rho: 0.524, n = 55, p < .001).


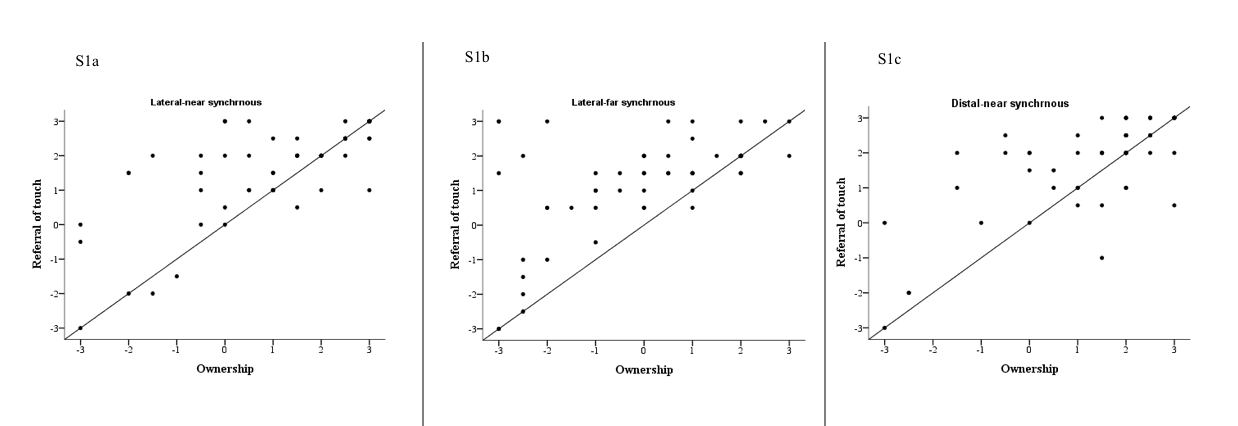


**Fig S1:** Scatter plots of correlation between ownership and referral of touch during synchronous stimulation. (S1a) Lateral-near (S1b) Lateral-far (S1c) Distal-near.

# Experiment 2 - Ownership vs referral of touch related questions

Here again, the association between ownership and referral of touch scores were examined using Spearman’s rho correlation analyses. Correlations between the two scores were found in all four positions (see Fig S2): distal-near (Spearman’s rho: -0.544, n = 44, *p* < .001), Distal-far (Spearman’s rho: -0.671, n = 44, *p* < .001), distal-very far (Spearman’s rho: -0.468, n = 44, p = .001), and the lateral-near (Spearman’s rho: -0.662, n = 44, *p* < .001)


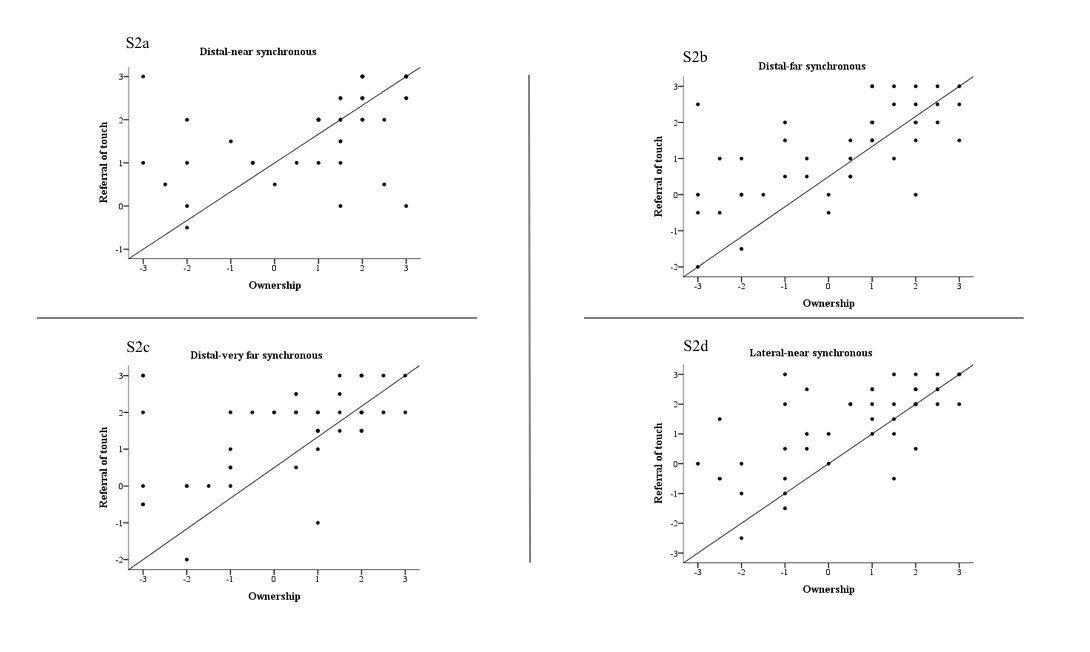


**Fig S2:** Scatter plots of correlation between ownership and referral of touch during synchronous stimulation. (S2a) Distal-near (S2b) Distal-far (S2c) Distal-very-far and (S2d) Lateral-near.

References

Longo, M. R., & Haggard, P. (2012). Implicit body representations and the conscious body image. *Acta Psychologica*, *141*(2), 164–168. <https://doi.org/10.1016/j.actpsy.2012.07.015>
